# A Simple Means of Making Carbon Dioxide Snow

**Published:** 1914-04-11

**Authors:** C. Thackray Parsons


### A Simple Means of Making Carbon Dioxide Snow.

*To the Editor of* The Hospital.

Sir,—In your review last week of Dr. Hall-Edwards' book on "Carbon Dioxide Snow" you referred to the elaborate character and cost of the special outfits usually recommended for making the snow. There is, however, a simple method, first described by Tousey, which is easy to carry out and suitable for those medical men who require only small quantities. All that is required is a small cylinder of liquid $CO_2$ fitted with a nozzle about two inches long. A piece of ordinary thick blotting-paper is taken and wound tightly round a rod of about the same calibre as the nozzle. One end of the paper should project about two inches beyond the rod. This end is folded upon itself so as to close completely the tube of blotting-paper. The fold is held in position by a bandage applied firmly over the paper and the rod in such a way that two free ends are left at the open end of the tube. The rod is then removed from the tube of paper and the tube slipped over the nozzle of the cylinder, to which it is fixed by tying the free ends of the bandage tightly. The stopcock of the cylinder is then opened and shut quickly four or five times so as to allow the liquid $CO_2$ to escape into the tube in gushes. The tube will be felt to grow cold and hard under the fingers as it becomes filled with the solid snow. When the tube is full of snow the gas will escape round the nozzle in the form of fine mist. The stopcock should then be turned off completely, the tube removed, the bandage unwound, and the cylinder of blotting-paper opened. Inside it will be found a stick of solid snow. This can be used as it is, or if required more compressed the stick can be broken up and the pieces placed inside a mould of the size and shape required and hammered to any hardness desirable.

Moulds of various shapes and sizes fitted with plungers to compress the snow can be bought very cheaply from most instrument makers. It is important that the cylinder of $CO_2$ should be held in such a way that liquid $CO_2$ and not the gas escapes when the tap is turned on. Most cylinders are made with a tube inside them running from the tap to the bottom of the cylinder. In that case the cylinder must be held with the nozzle pointing upwards when the tap is turned on. Sometimes, however, cylinders are supplied which do not contain this inside tube, and then the cylinder must be held with the nozzle pointing downwards. The only objection to this method is that a certain amount of $CO_2$ is wasted, but this is a matter of small importance when only a small quantity of snow is required.—Yours faithfully,

Fulham Infirmary. C. Thackray Parsons, M.D.

[*Note.—Owing to the pressure on our space we have had to hold over several letters this week.*]

